# Ultra-broadband enhancement of nonlinear optical processes from randomly patterned super absorbing metasurfaces

**DOI:** 10.1038/s41598-017-04688-4

**Published:** 2017-06-28

**Authors:** Nan Zhang, Ziheng Ji, Alec R. Cheney, Haomin Song, Dengxin Ji, Xie Zeng, Borui Chen, Tianmu Zhang, Alexander N. Cartwright, Kebin Shi, Qiaoqiang Gan

**Affiliations:** 1 Department of Electrical Engineering, The State University of New York at Buffalo, Buffalo, NY 14260 USA; 2 State Key Laboratory for Mesoscopic Physics, Collaborative Innovation Center of Quantum Matter, School of Physics, Peking University, Beijing, 100871 China

## Abstract

Broadband light trapping and field localization is highly desired in enhanced light-matter interaction, especially in harmonic generations. However, due to the limited resonant bandwidth, most periodic plasmonic nanostructures cannot cover both fundamental excitation wavelength and harmonic generation wavelength simultaneously. Therefore, most previously reported plasmonic nonlinear optical processes are low in conversion efficiency. Here, we report a strong enhancement of second harmonic generation based on a three-layered super absorbing metasurface structure consisting of a dielectric spacer layer sandwiched by an array of random metallic nanoantennas and a metal ground plate. Intriguingly, the strong light trapping band (e.g. >80%) was realized throughout the entire visible to near-infrared spectral regime (i.e., from 435 nm to 1100 nm), enabling plasmonically enhanced surface harmonic generation and frequency mixing across a broad range of excitation wavelengths, which cannot be achieved with narrow band periodic plasmonic structures. By introducing hybrid random antenna arrays with small metallic nanoparticles and ultra-thin nonlinear optical films (e.g. TiO_2_) into the nanogaps, the nonlinear optical process can be further enhanced. This broadband light-trapping metastructure shows its potential as a building block for emerging nonlinear optical meta-atoms.

## Introduction

Nonlinear light-matter interaction at mesoscopic scales has emerged as an intriguing platform for studying fundamental optical physics and developing practical photonic applications^1, 2^. However, the general light-matter interaction efficiency of many conventional nonlinear optical processes is low^3, 4^. Therefore, high intensity lasers are usually required in conventional nonlinear optical experiments. As a result, plasmonics, which leverages the resonant interaction between light and free electrons of metallic nanostructures, has garnered interest as a means to confine electromagnetic fields within nanoscale volumes and significantly enhance localized field^5, 6^. For instance, periodic patterns were utilized in three-layered metamaterial super absorbers that were reported to enhance the second harmonic generation (SHG) signal with unique electronic tunability^7^. However, periodic nanostructures and other specifically designed nanopatterns rely on top-down lithographic techniques (e.g. focused ion beam milling^8, 9^, electronic-beam lithography^10, 11^, nanoimprint lithography^12^ and combined hybrid fabrication methods^13, 14^), imposing serious cost barriers for practical applications. In addition, according to the definition of the enhancement factor (i.e., ${\rm{EF}}={(\frac{{{\rm{E}}}_{{\rm{local}}}({\rm{\omega }})}{{{\rm{E}}}_{0}({\rm{\omega }})})}^{2}{(\frac{{{\rm{E}}}_{{\rm{local}}}(2{\rm{\omega }})}{{{\rm{E}}}_{0}(2{\rm{\omega }})})}^{2}$ ^15^), an ideal structure to enhance the SHG is to enhance the localized field at excitation and emission wavelengths simultaneously within the same surface structure. Unfortunately, due to the narrow resonant nature of periodic structures, the enhanced spectral range is usually limited, that is insufficient to cover both excitation and emission wavelengths. Thus, broadband light trapping and localization are essential in the development of practical on-chip optoelectronic circuits that leverage nonlinear optical processes in their operation.

To address this challenge, a simple, low-cost, scalable, lithography-free method to manufacture super-absorbing metasurfaces was recently developed^16^. By controlling the average geometric parameters of directly deposited random metal nanoparticles (NPs) with a low degree of symmetry, a super-absorbing plasmonic metamaterial structure was realized with broadband resonant spectral tunability (e.g. >80% absorption band from 414 nm to 956 nm). In this case, when the strongly trapped light interacts with these random nanoantennas, it resonantly couples to free-electrons at the edges of NPs with the appropriate geometry. We have recently shown that a single piece of such a “metasurface” can be used as a universal substrate for excitation wavelengths (λ_ex_) lying within its light trapping band for applications that leverage surface enhanced light-matter interaction, specifically surface-enhanced Raman spectroscopy (SERS)^17^. According to our previously reported experiment to resolve chemical molecules, an enhancement factor over 10^7^ was obtained. It should be noted that both SERS and plasmonically enhanced nonlinear optical effects share similar requirements in terms of strong local field enhancement at both excitation and emission wavelengths^5^. In particular, nonlinear optics require an even broader light trapping band: e.g. dual resonant SHG requires the localized field enhancement from λ_ex_/2 to λ_ex_, which is significantly broader than the required spectrum for SERS near a given λ_ex_. Therefore, broadband light-trapping metastructures will also behave as a novel platform for plasmonically enhanced surface harmonic generation and frequency mixing across a broad range of excitation wavelengths, which has not yet been achieved with narrow band periodic plasmonic structures. In this work, we report ultra-broadband SHG using metasurfaces engineered with random NP arrays, which show promise as a building block for emerging nonlinear optical meta-atoms^6, 7, 18^.

## Results and Discussion

### Ultra-broadband super absorbing metasurfaces

**Figure 1:**
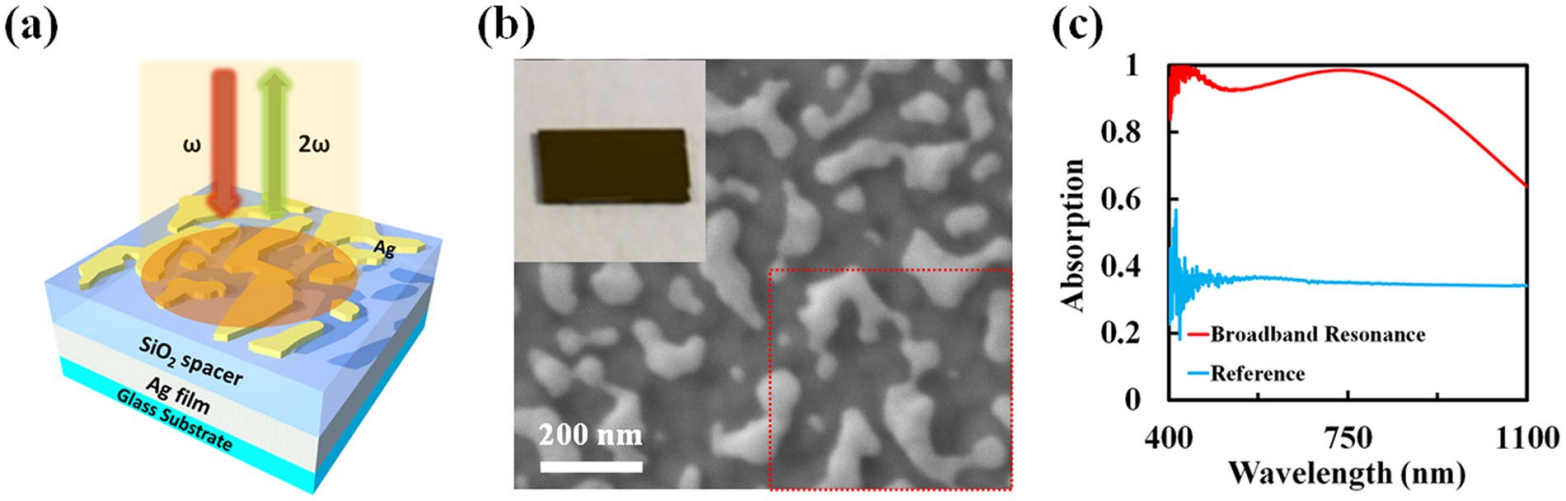
(**a**) Schematic of the designed three-layered absorbing metasurface. (**b**) SEM image of top random Ag NPs. Inset: Photograph of the ultra-broadband super absorbing metasurface. (**c**) Absorption spectra of the three-layered absorber (red curve) and the reference structure (blue curve).

As illustrated in Fig. 1(a), our three-layered random metal-dielectric-metal (rMDM) super absorbing metasurface consists of a 200-nm-thick silver (Ag) film as a reflector, a 70-nm-thick silicon dioxide (SiO_2_) spacer layer and a layer of Ag random NPs. Following our previously reported lithography-free fabrication technique^16^, direct Ag deposition followed by thermal annealing was used to manipulate the average morphology (size, spacing, and “interconnectedness”) of the Ag NPs to tune the effective optical constant and realize the desired light-trapping band (see Methods for details of fabrication). In this experiment, the Ag NPs were formed by annealing a 14-nm-thick Ag film under 200 °C for 1 hour. The morphology of this sample was characterized by scanning electronic microscope (SEM) as shown in Fig. 1(b), indicating that randomly distributed Ag NPs were directly obtained during the thermal annealing process. The inset of Fig. 1(b) shows a color photograph of the sample, where one can note the almost black appearance of the highly broadband absorbing metasurface. The optical absorption of the metasurface was characterized using a Fourier transform infrared spectrometer (FTIR, Bruker VERTEX 70) with an extended light source covering visible wavelengths. As shown by the red curve in Fig. 1(c), a strong resonant absorption peak of 98.6% was obtained at the wavelength of 736 nm with the 80% absorption band spanning from 400 nm to 980 nm, which is significantly broader than the previous report^19^. For reference, we used a single Ag NP layer on glass substrate with no SiO_2_ spacer or Ag film. The reference optical absorption spectrum is shown by the blue curve in Fig. 1(c). One can see that the absorption of the metasurface at 736 nm is over 98% while the reference sample is only 35%, indicating the enhanced light trapping in the metasurface structure. When the incident light is trapped in the metasurface, it is re-distributed between the random NPs, which results in significantly enhanced local fields for surface enhanced nonlinear optics. It should be noted that the morphologies of rMDM metasurface and the reference sample are similar due to the similar wettabilities at the interface of Ag/SiO_2_-dielectric-layer and Ag/glass-substrate (see Section 1 in the supporting material for statistical analysis of the NP size distribution).

### Nonlinear optical response of broadband metasurfaces

**Figure 2:**
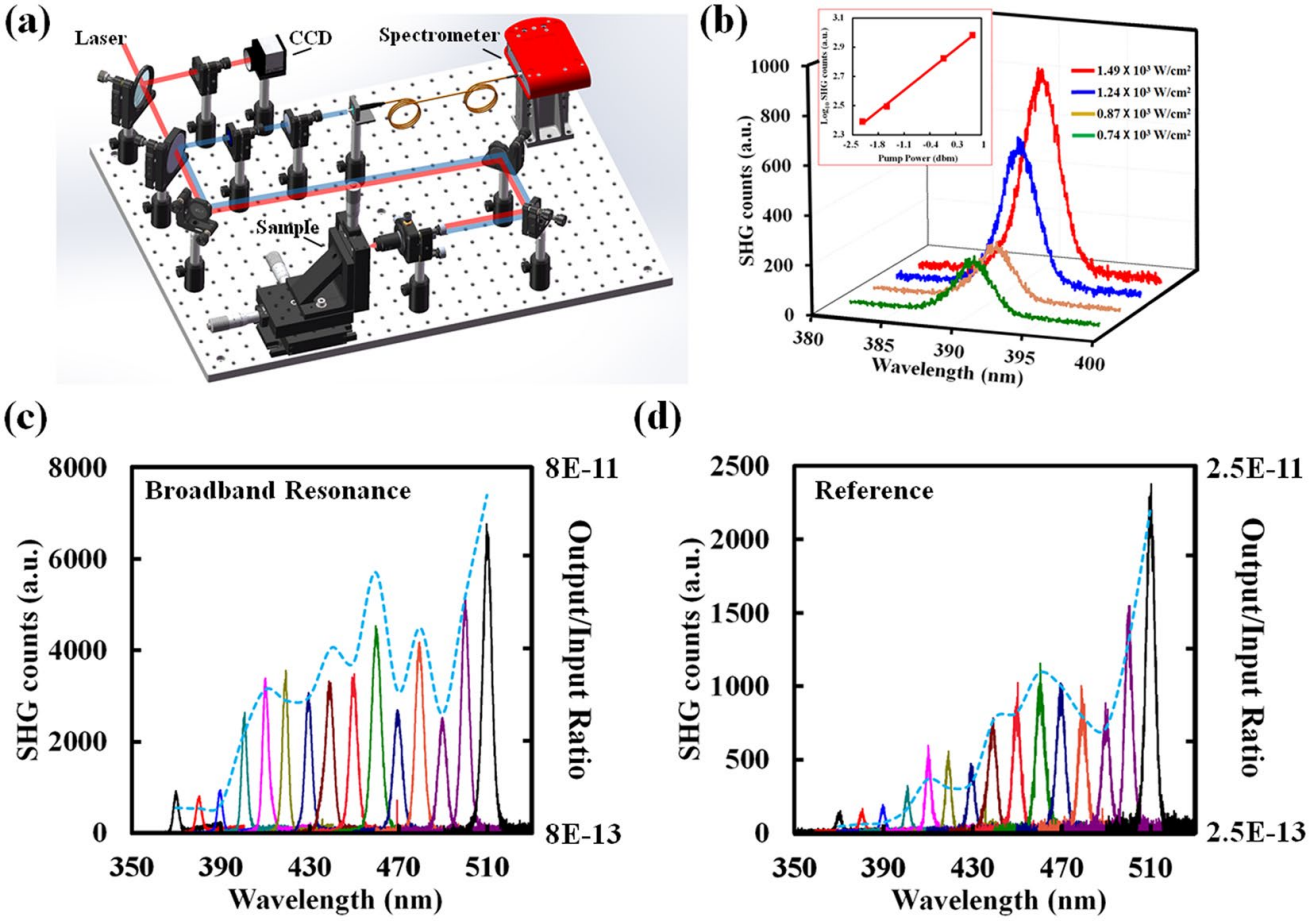
(**a**) Schematic of experimental setup for SHG measurement of fabricated metasurfaces. (**b**) Measured SHG intensities at different incident power. The inset is a log-log plot of amplitude of the SHG signal as a function of incident power. (**c**,**d**) Measured SHG intensities and SHG output/input ratios (blue dashed curve) versus incident wavelengths obtained with (**c**) the broadband metasurface and (**d**) the reference sample, respectively.

To demonstrate the enhancement of SHG in the metasurface, we studied the nonlinear optical properties of the random metasurface with the setup shown schematically in Fig. 2(a). The sample was irradiated with an ultrafast laser tunable from 740 nm to 1020 nm, with the pulse length of 80 fs and a repetition rate of 80 MHz, generated by using a broadband Ti-sapphire oscillator (Spectra-Physics MaiTai) and an optical parametric oscillator (Spectra-Physics Inspire 100) (see Methods for details of SHG experimental setup). The average power of the fundamental beam was controlled by a continuously variable neutral density filter and set to 1.2 mW as measured at the sample position to avoid damage to the top NPs. The second harmonic signal was then collected in reflection mode and analyzed by a spectrometer (Acton SpectraPro, SP-2500) equipped with a liquid nitrogen cooled charge-coupled device (CCD). The diameter of the laser spot is approximately 8 µm. Figure 2(b) shows the emission spectra generated using a focused 780 nm fundamental excitation beam with different average powers ranging from 0.74 × 10^3^ W/cm^2^ to 1.49 × 10^3^ W/cm^2^. An obvious signal at 390 nm was observed. The inset of Fig. 2(b) is a log-log plot of signal amplitude as a function of incident power, which exhibits a clear linear dependence with a slope of ~2.02. This corresponds to a quadratic dependence of signal on incident power, thus confirming the measured signals are from SHG (i.e., proportional to |E|^2^).

Importantly, the broadband light-trapping of the metasurface with random nanoantennas should likewise result in a unique broadband SHG. To validate this broadband nonlinear optical response, we then leveraged the tunability of the ultra-fast laser to excite the same chip with different fundamental wavelengths. As shown in Fig. 2(c), when the excitation wavelength was tuned from 740 nm to 1020 nm, obvious SHG signals from 370 nm to 510 nm were observed from the broadband metasurface (see Section 2 in the supporting material for details of SHG data calibration). In contrast, the reference sample (Fig. 2(d)) exhibited much smaller SHG intensities with the same input power, indicating an enhancement of light-matter interaction in the rMDM broadband super-absorbing metasurface.

### Plasmonic mode matching in the random super absorbing metasurface

**Figure 3:**
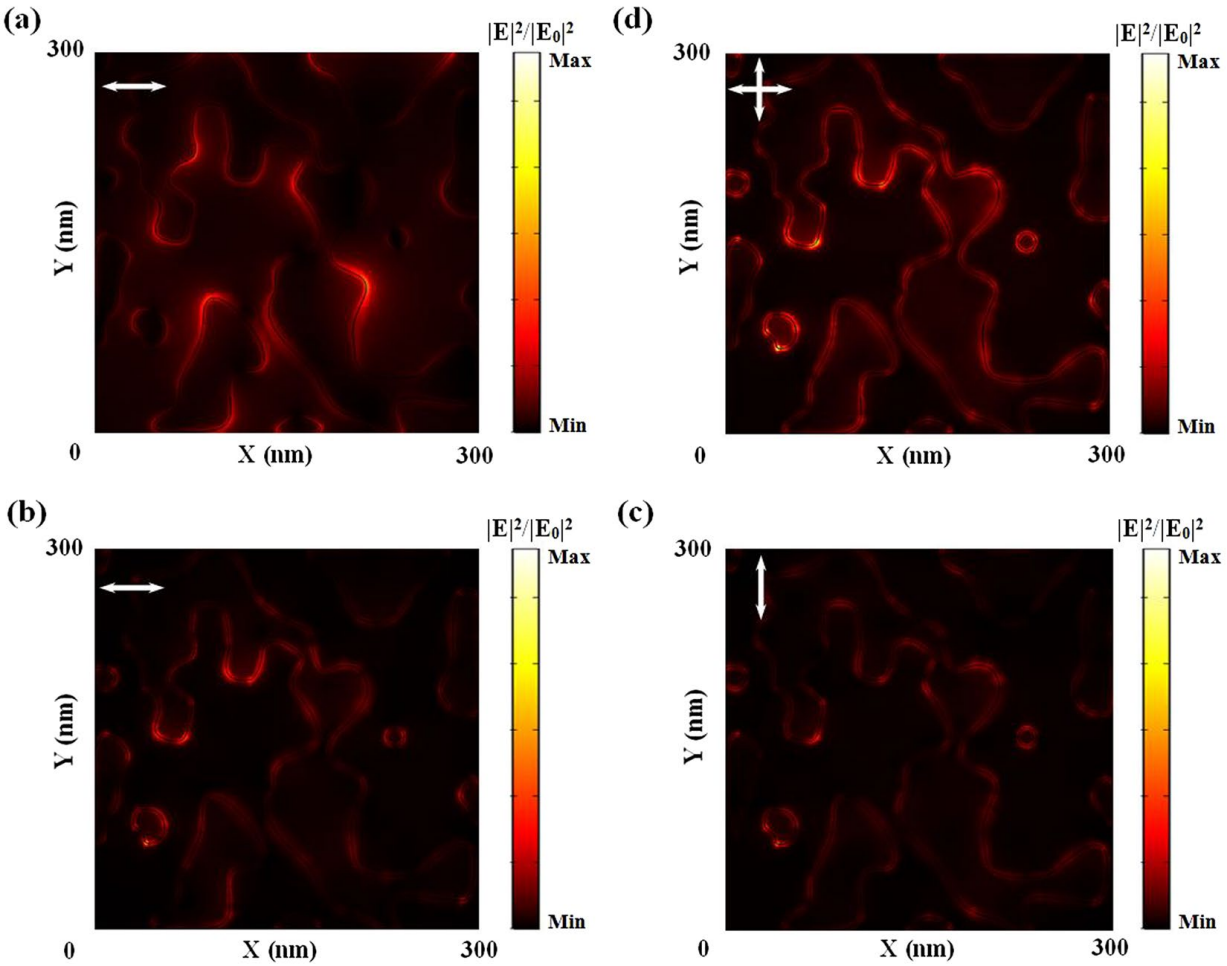
Modeled electric field enhancement distribution among NPs in the red dotted squares shown in Fig. 1(b) at 1000 nm wavelength with x-polarization (**a**), at 500 nm wavelength with x-polarization (**b**), at 500 nm wavelength with y-polarization (**c**), and at 500 nm wavelength with x- and y-polarization (**d**).

In most periodically patterned metallic structures for plasmonic nonlinear optics, spatial overlap of plasmon modes was typically not emphasized since highly periodic structures produce narrow resonance bands (e.g. refs 20–25). According to a recent report, sophisticated nanostructures were employed to realize two resonances at λ_ex_/2 and λ_ex_ simultaneously with highly overlapped spatial mode distributions to improve the SHG efficiency^26^. In our broadband rMDM structure, the light trapping at λ_ex_/2 and λ_ex_ is addressed satisfactorily. The remaining question is the spatial mode overlap. In random structures, depolarization introduced by hyper-Reyleigh scattering from random NPs will result in incoherency of SHG signals^27–31^. It is therefore difficult to manipulate the spatial mode distribution. Recently, a cavity was introduced to enhance the electric field at both fundamental and SHG wavelengths, and realized a spectral and spatial coincidence of SH response with the free excitons of ZnO nanorods^32^. It also indicates that an overlapped spatial mode is highly desired to improve the SHG efficiency. To interpret the mode overlap in our rMDM structure, we loaded a part of the SEM image in Fig. 1(b) (i.e., the area within the red dotted square) into the commercial software COMSOL, and modeled the spatial distribution of the electric field. The localized field distribution at the wavelength of 1000 nm under a linearly polarized excitation is shown in Fig. 3(a). In our experiment, we employed a polarizer to characterize the polarization dependence and confirmed that the SHG signal is non-polarized (see Section 3 in the supporting material for raw data). Therefore, the localized field distribution at 500 nm is the summation of x- (Fig. 3(b)) and y-polarized situation (Fig. 3 (c)), as shown in Fig. 3(d). To evaluate the mode matching situation, we calculated the correlation between Fig. 3(a and b–d). The correlation between these two mode distributions is 24.0% (calculated by comparing Fig. 3(a) with Fig. 3(b)), 21.4% (calculated by comparing Fig. 3(a) with Fig. 3(c)), and 37.9% (calculated by comparing Fig. 3(a) with Fig. 3(d)), respectively. Therefore, the depolarization effect for the SHG signal is actually beneficial with respect to mode matching requirements.

### Further enhancement of SHG

**Figure 4:**
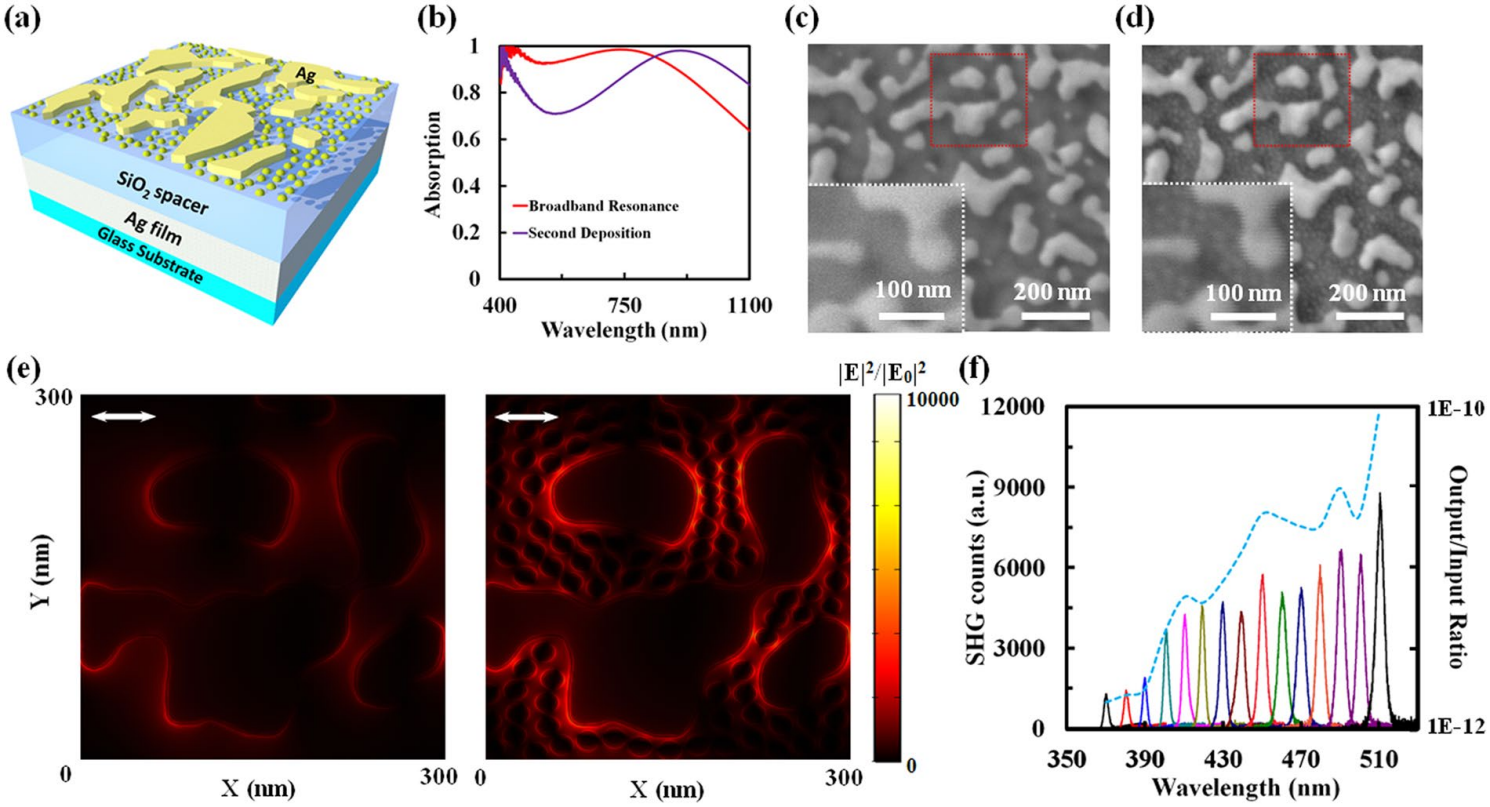
(**a**) Schematic of the designed three-layered absorbing metasurface after a multi-step deposition process. (**b**) Absorption spectra of the three-layered absorber before (red curve) and after the second-step deposition (purple curve). (**c**,**d**) SEM images of top random Ag nanoparticles (**c**) before and (**d**) after an extra 5-nm-thick NPs deposition. The scale bar is 200 nm. Red dotted squares: areas loaded for simulation. White dotted square: zoom-in SEM images of the surface morphology before and after the second-step NP deposition. (**e**) Modeled electric field enhancement distribution among NPs in the red dotted squares in (**c**) and (**d**) at normal incidence at λ = 1000 nm, polarized in the x direction. (**f**) Measured SHG intensities and SHG output/input ratios (blue dashed curve) versus excitation wavelengths obtained with the broadband metasurface after a multi-step deposition process.

It is generally believed that smaller gaps between metallic nanopatterns will result in stronger localized field due to optically driven free electrons coupled across the gap. In recent years, significant effort has been invested to reveal the upper limit for plasmonic enhancement using ultra-small gaps (e.g. refs 33–36), even approaching the quantum limit within subnanometer regions^37–42^. A straightforward subsequent question is how we can reduce the gap between our random NPs to further enhance the effective nonlinearity of the metasurface and their corresponding SHG signals. In using thermal annealing to control the morphology and size of surface NPs, it was observed that the spacing between adjacent NPs is also controllable. During the annealing process, metal atoms in the film migrate and aggregate to form islands^43^, and the void left in their absence defines the interparticle spacing. The more atoms aggregate, the larger interparticle spacing will form. Thus it can be seen that the spacing between smaller NPs is generally smaller than the spacing between larger NPs. With this in mind, we will discuss another multi-step direct deposition method to realize smaller gaps, as illustrated in Fig. 4(a). Starting with samples fabricated as described above, we then deposited a second Ag film, 5 nm thick to be below the percolation threshold of ~6 nm^44^, on top of the random nanopattern that results from the aforementioned annealing process. Optical characterization, as shown by the purple curve in Fig. 4(b), revealed a red shift in the absorption peak from 736 nm to 908 nm due to multi-mode resonances^45^. A strong broadband absorption was maintained, with >80% absorption bands both from <400 nm to 472 nm and from 686 nm to 1100 nm. Comparing SEM images before and after this second deposition (Fig. 4(c) and (d), respectively) confirmed that smaller NPs were formed in the area between large metal islands as expected. The average gap distances between NPs in this case are reduced to below 10 nm, and therefore should support stronger localized field. To validate this prediction, we loaded a part of the SEM image of the top films (see the red dotted square) to model the spatial distribution of the electric field at normal incidence at λ = 1000 nm, with x-polarization. As shown in Fig. 4(e), more hot spots are obtained between large and small NPs, and with stronger localized field intensities. The incident light is mainly localized at edges of Ag islands, which is the major mechanism for the proposed SHG enhancement (see Section 4 in the supporting material for detailed analysis of electric field distribution). We then characterized the SHG of the same area on the sample. As shown in Fig. 4(f), the broadband super absorbing metasurface after a second-step deposition process exhibits much stronger SHG intensities, indicating a further enhanced light-matter interaction by introducing smaller gaps over a larger area.

### Enhanced SHG with TiO_2_ films

**Figure 5:**
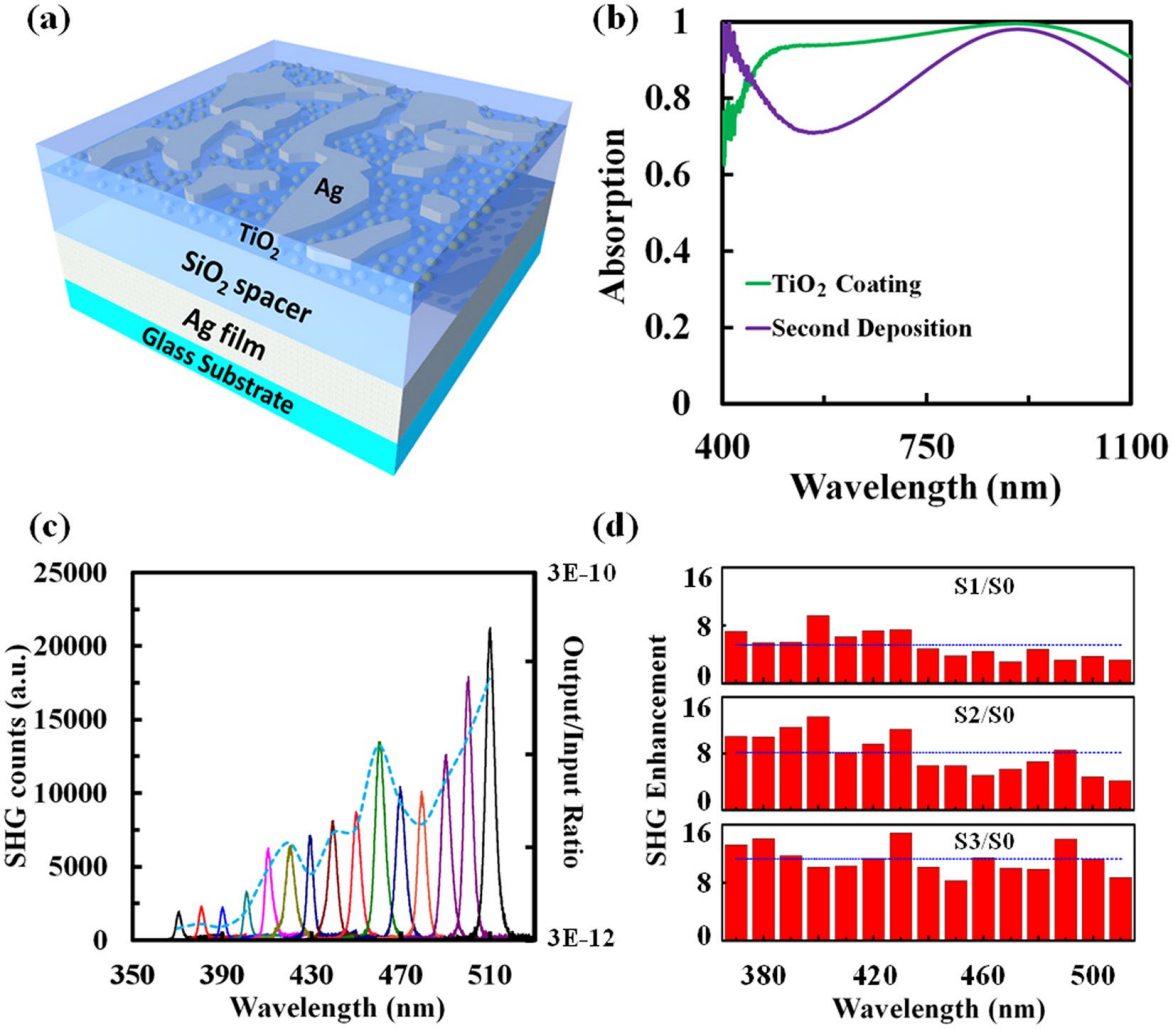
(**a**) Schematic of the designed three-layered absorbing metasurface after the multi-step deposition and TiO_2_ film coating. (**b**) Absorption spectra of the three-layered absorber with a second-step deposition process before (purple curve) and after TiO_2_ film coating (green curve). (**c**) Measured SHG intensities and SHG output/input ratios (blue dashed curve) versus incident wavelengths obtained with the broadband metasurface after the multi-step deposition and TiO_2_ film coating. (**d**) Spectroscopic enhancement of SHG from the broadband metasurface S1, the metasurface after the fine-particle deposition S2, and the metasurface after fine-particle deposition and TiO_2_ film coating S3 compared with that from the reference sample S0. Blue dotted lines: The averaged SHG enhancement over the entire wavelength region.

Finally, by integrating an optically transparent material with strong optical nonlinearity into the super absorbing metasurface with the fine particle deposition, SHG signals are expected to be further enhanced due to the combined effects of both the nonlinear material and localized field enhancement from optical nanoantennas on the metasurface. Titanium dioxide (TiO_2_) was chosen for this purpose since it has a high nonlinear refractive index that is 30 times that of silica^46^, while still being transparent in the visible and NIR spectral regions due to its large band gap of 3.1 eV^47^. We employed atomic layer deposition to deposit a 14 nm TiO_2_ film at 150 °C to cover the large and small NPs on the metasurface (see Methods for details of fabrication), as illustrated in Fig. 5(a). High temperatures can facilitate the crystallization of TiO_2_ into the polycrystalline-anatase phase^48^. Therefore, the super absorbing metasurface with the TiO_2_ film was then thermally annealed at 400 °C for 1 hour. As shown by the green curve in Fig. 5(b), a broader and higher absorption was obtained with >80% absorption from 435 nm to 1100 nm (or >90% absorption from 466 nm to 1100 nm). This enhancement in absorption should be attributed to the resonance of the three-layered rMDM super absorbing metasurface, which is sensitive to the dielectric environment^49^: when we coated the top metallic NPs with a thin film TiO_2_ layer, its dielectric environment was changed. Therefore, although the TiO_2_ does not absorb visible and IR light, the resonant condition was changed. The light trapping and optical absorption still occurred within the metallic NP layer.

It is well-known that the symmetry of materials is inherently broken at the surface. Due to the anisotropy of the electric field at the interface, the potential landscape experienced by an atom at the interface is non-symmetric, leading to a surface mediated second-order susceptibility^5, 50^. Thus, for a thin film TiO_2_ over metal islands (a system with a high interfacial area), significant SHG occurs at these metal-TiO_2_ interfaces. Although the morphology of metasurface changed during the final thermal annealing process for TiO_2_ (see discussion in Section 5 in the supporting material), this additional thin TiO_2_ layer facilitates much stronger SHG intensities, indicating a further enhanced light-matter interaction by introducing nonlinear materials, as shown in Fig. 5(c). For clarity, Fig. 5(d) shows a comparison of spectroscopic enhancement of SHG from different samples compared with the reference sample (i.e., a single layer of deposited NPs on the glass substrate). The reference sample, the broadband metasurface, the metasurface after the fine-particle deposition, and the metasurface after both fine particle deposition and TiO_2_ film coating are denoted as S0, S1, S2, and S3, respectively. This comparison clearly demonstrates the improved nonlinear optical properties of the broadband metasurface structure by introducing smaller gaps and TiO_2_ films. Furthermore, the measured SHG output/input ratio (defined as the measured P(2ω)/P(ω)) of sample S3 at an excitation wavelength of 1020 nm is 2.14 × 10^−10^ with a pumping intensity of only 6 kW/cm^2^. In comparison, previously demonstrated nonlinear optical metasurfaces at infrared/visible/ultraviolet wavelengths required a much higher peak power intensity to produce an SHG conversion efficiency of only 10^−11^ ^7, 21, 51^. Even though some work reported higher SHG efficiency of up to 10^−9^, the top-down lithographic techniques used in these works still impose serious cost barriers for practical applications^24, 52, 53^.

## Conclusion

In conclusion, we reported a scalable, low cost and broadband super absorbing metasurface substrate for strong field localization and enhanced surface enhance nonlinear optical processes. By manipulating the morphology and composite of the top random nanoantenna layer, an ultra-brandband light trapping was experimentally demonstrated in the wavelength range from 435 nm to 1100 nm with strongly localized field. This new plasmonic nonlinear light generation structure has achieved high SHG enhancement by introducing a second-step metal particle deposition process and a nonlinear TiO_2_ film, compared with the reference sample of only Ag NPs on a bare glass substrate. Importantly, this broadband light trapping metasurface structure is completely lithography free, suitable for future large area roll-to-roll deposition processes for inexpensive nanomanufacturing. In addition to providing new understanding of broadband light trapping and field localization, this work may open avenues toward new applications in energy harvesting^20, 54–56^, conversion^57–60^ and surface enhanced Raman spectroscopy^61–63^.

## Methods

### Metasurface Fabrication

2.5 × 7.5 cm^2^ microscope glass slides were sequentially sonicated in acetone, isopropyl alcohol (IPA), and deionized water for 15 minutes. The fabrication of metal/dielectric/metal metasurface began with a 200-nm-thick Ag ground plate deposited on the glass slide using AJA sputtering/e-beam evaporation dual chamber hybrid thin film deposition system. During the deposition, the Argon pressure in the chamber was set to 3 mTorr with no heating on the substrate. The Ag deposition rate was controlled by the sputter voltage and direct current power at 2.6 Å/sec. Then the 70 nm SiO_2_ film was deposited using an electron-beam evaporation system (BOC Edwards Auto 500 system) with the deposition rate of 1 Å/sec under vacuum (7.0 × 10^−6^ Torr). For the top layer, 14 nm-thick Ag film was deposited with the deposition rate of 2.6 Å/sec in the chamber of AJA sputtering system. Next, the Ag mesh network was transformed to isolated nanoparticles by introducing a thermal annealing process at the temperature of 200 °C (i.e., Fig. 1(b)) for 60 minutes. In the second-step direct deposition process, a 5 nm-thick Ag film was deposited with a deposition rate of 2.6 Å/sec in the chamber of AJA sputtering system. Finally, a 14 nm-thick TiO_2_ film was deposited using Ultratech/CambridgeNanotech atomic layer deposition system at 150 °C.

### Characterization

SEM images were taken using Zeiss CrossBeam® Workstation system. The reflection/absorption spectra of metasurfaces were characterized using a microscopic Fourier transform infrared spectroscopy (Bruker, VETEX 70 + Hyperion 1000) and UV/Vis/NIR spectrophotometer (Perkin-Elmer Lambda 750). The observation area for each sample was set to 50 μm × 50 μm.

### SHG experimental setup

Laser pulses (red beam in Fig. 2(a)) were generated by using a broad-band Ti:sapphire oscillator (Spectra-Physics MaiTai) and an optical parametric oscillator (Spectra-Physics Inspire 100) with 80 fs duration and 80 MHz repetition rate, then passed through a long pass filter (ET542lp Chroma) before being directed into a home-made upright microscope. The laser beam was vertically focused on the metasurface by a CaF2 UV objective (LMU-15X-UVB NA0.32 Thorlabs). The fundamental wavelength was scanned from 740 nm to 1020 nm with 20 nm increments. The SHG signal (blue beam in Fig. 2(a)) was collected in the backward direction, extracted by a long pass dichroic mirror (DMLP567 Thorlabs, 340dclp Chroma) and filters (D350-50x/ET385-70X/ET700sp Chroma). A spectrograph (Acton SpectraPro, SP-2500) equipped with a liquid nitrogen cooled CCD is used to analyze the SHG spectra. The pump power of the fundamental beam at all wavelengths was set to 1.2 mW as measured at the sample position. SHG spectra were measured by scanning the fundamental wavelengths.

## Electronic supplementary material

Supplementary Information
