# Supplementary material for: Ultra-broadband enhancement of nonlinear optical processes from randomly patterned super absorbing metasurfaces
Source: Sci Rep. 2017 Jun 28;7:4346. doi: 10.1038/s41598-017-04688-4 (PMC5489484; doi:10.1038/s41598-017-04688-4)
Supplement: Supplementary file 1 — Supplementary Information [file 41598_2017_4688_MOESM1_ESM.doc]

**Supplementary Information:**

**Ultra-broadband enhancement of nonlinear optical processes from randomly patterned super absorbing metasurfaces**

Nan Zhang^1,*^, Ziheng Ji^2,*^, Alec R. Cheney^1,*^, Haomin Song^1^, Dengxin Ji^1^, Xie Zeng^1^,

Borui Chen^1^, Tianmu Zhang^1^, Alexander N. Cartwright^1^, Kebin Shi^2,†^, Qiaoqiang Gan^1,‡^

*^1^Department of Electrical Engineering, The State University of New York at Buffalo, Buffalo, NY 14260, U.S.A*

*^2^ State Key Laboratory for Mesoscopic Physics, Collaborative Innovation Center of Quantum Matter, School of Physics,*

*Peking University, Beijing 100871, China*

* These authors contribute equally to this manuscript.

† [kebinshi@pku.edu.cn](mailto:kebinshi@pku.edu.cn); ‡ [qqgan@buffalo.edu](mailto:qqgan@buffalo.edu)

1. **SEM images of the rMDM super absorbing metasurface and the reference sample**

The SEM images of NPs on the rMDM super absorbing metasurface and the reference sample are shown in Figs. S1(a) and S1(b), respectively. One can see similar surface morphologies on these two samples due to the similar wettabilities at the interface of Ag/SiO_2_-dielectric-layer and Ag/glass-substrate. As shown in Figs. S1(c) and S1(d), the statistical analysis of the nanoparticles size distribution also confirmed similar morphologies of NPs on these two samples.


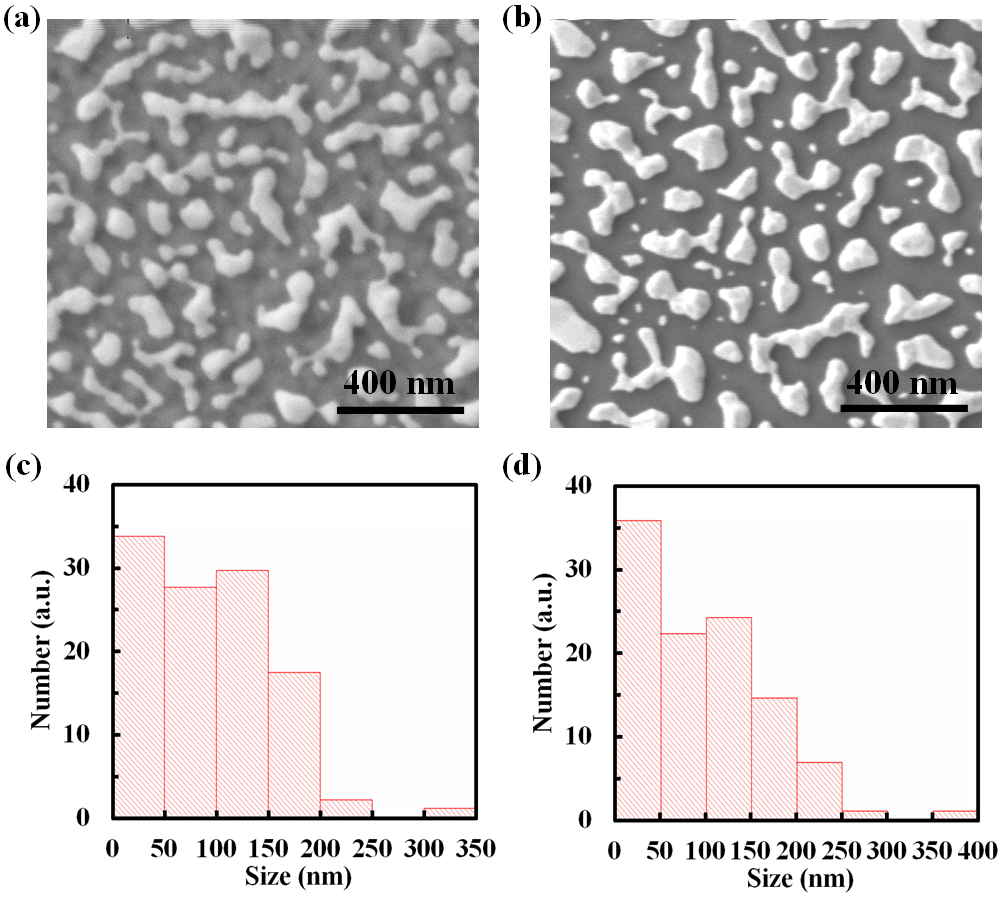


Figure S1. (a-b) SEM images of (a) rMDM super absorbing metasurface and (b) reference on the glass substrate. (c-d) Histograms of the nanoparticles size distribution of (c) metasurface and (d) reference.

1. **Second Harmonic Generation data calibration**

Before the SHG signal being received by the CCD, the signal went through various optical elements, including an objective, three aluminum mirrors, a dichroic mirror, a UV lens, a grating, and ultimately the CCD. Due to their wavelength-dependent transmittance, reflectance, diffraction and quantum efficiency (Fig. S2(a)), this effect cannot be neglected in our broad band measurement. Therefore, the raw data are divided by an overall efficiency (Fig. S2(b)) to recover the real SHG intensity profile.


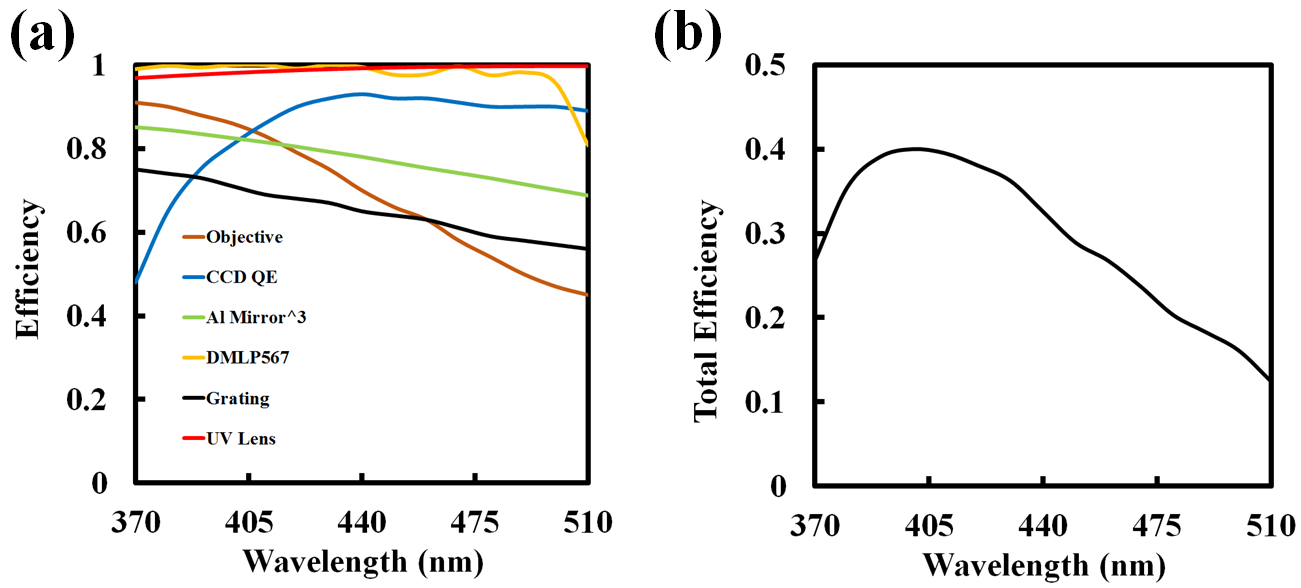


Figure S2. (a) Efficiency of different optical elements in the experimental setup. (b) The overall conversion efficiency of the experimental system over a broad spectral range.

1. **Experimental polarization dependence of the output SHG intensity**


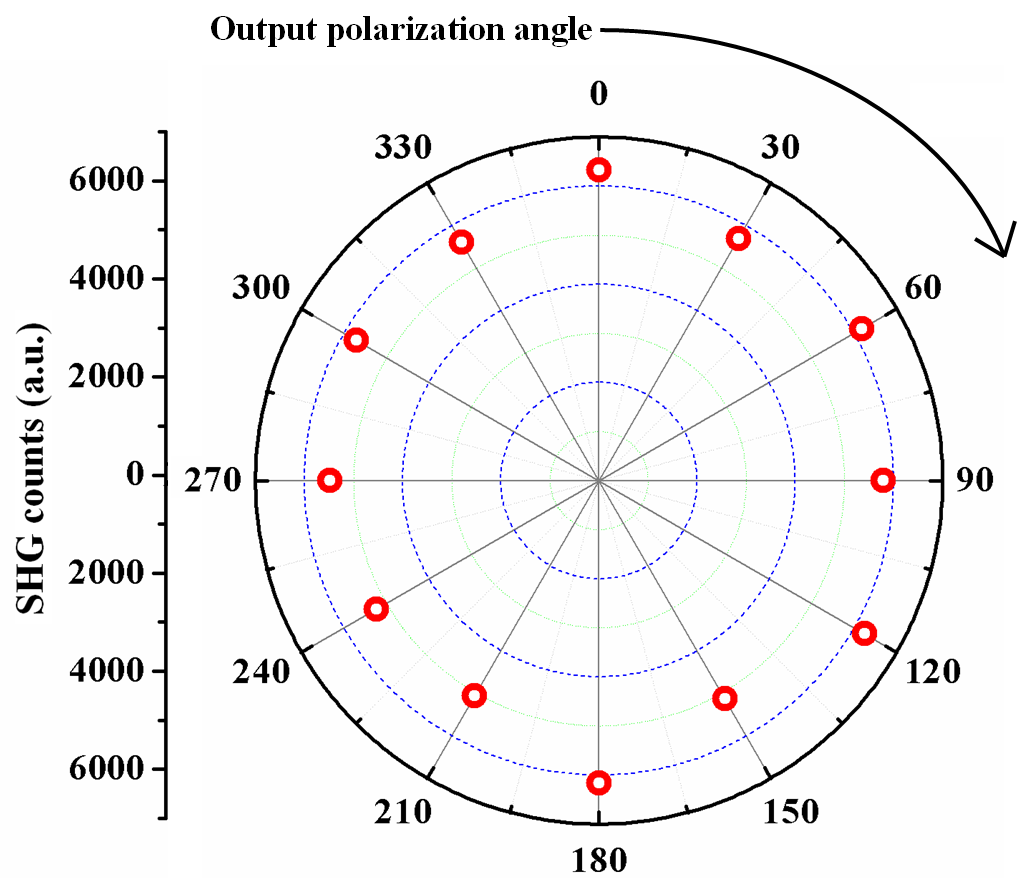


Figure S3. Polar plot of the output SHG intensity as a function of the output polarization angle.

1. **Surface and cross-sectional electric field distribution**

As shown in Fig. S4, the incident light is mainly localized at edges of Ag islands, which is the major mechanism for the proposed SHG enhancement.

**
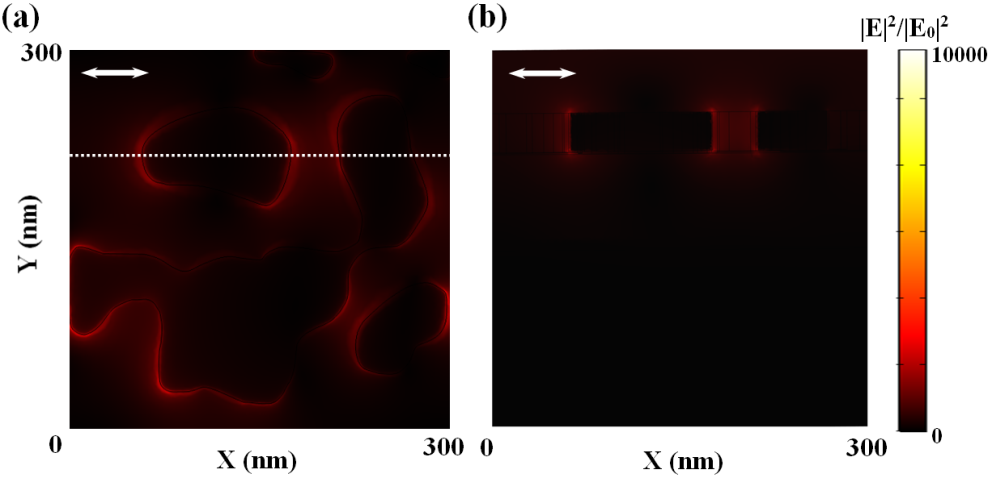
**

Figure S4. Modeled (a) surface and (b) cross-sectional electric field enhancement distribution among top random Ag nanoparticles. White dotted line in (a): section for cross-sectional electric field simulation in (b).

1. **SEM images of the rMDM super absorbing metasurface with extra small NPs before and after thermal annealing**

As shown in Fig. S5, after thermal annealing at 400 °C for 1 hour, high temperature induced the grain growth of the second-deposited small NPs. The initial large Ag islands became more rounded. Thus it can be seen that the sub-10nm gap distance introduced by the multi-step deposition increased during the annealing process. According to the further enhanced SHG with TiO_2_ coating as shown in Fig. 5(d) in the main text, the enhancement can be attributed to the introduction of the dielectric TiO_2_ material. Therefore, the extra TiO_2_ coating played a major role in the SHG enhancement.


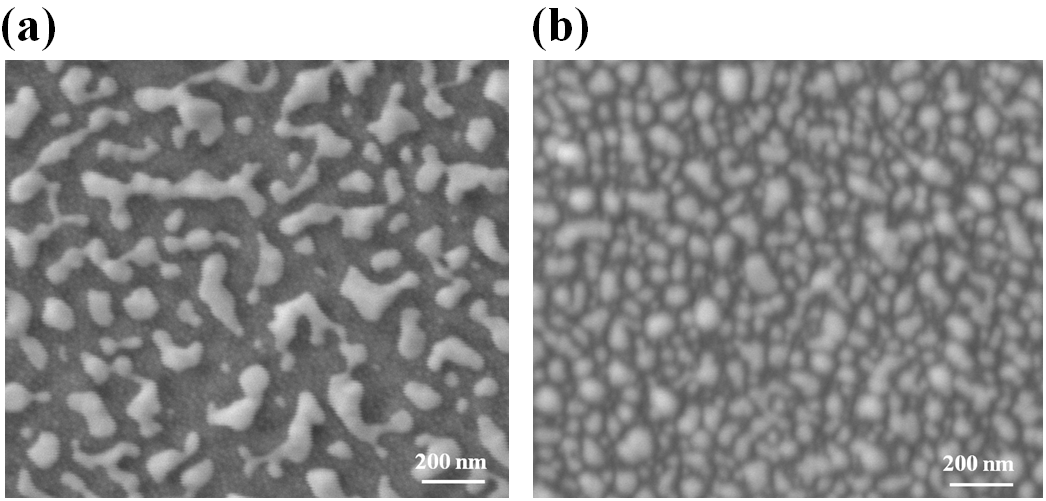


Figure S5. SEM images of the rMDM super absorbing metasurface (a) after the multi-step deposition and (b) TiO_2_ film coating and thermal annealing.
